# How do local authority plans to tackle obesity reflect systems thinking?

**DOI:** 10.1177/17579139221106337

**Published:** 2022-07-07

**Authors:** R Taheem, K Woods-Townsend, W Lawrence, J Baird, KM Godfrey, M Hanson

**Affiliations:** NIHR Southampton Biomedical Research Centre, University Hospital Southampton NHS Foundation Trust, Southampton, UK; Institute of Developmental Sciences, University of Southampton, Southampton, UK; NIHR Southampton Biomedical Research Centre, University of Southampton and University Hospital Southampton NHS Foundation Trust, Southampton, UK; Southampton Education School, Faculty of Social Sciences, University of Southampton, Southampton, UK; NIHR Southampton Biomedical Research Centre, University of Southampton and University Hospital Southampton NHS Foundation Trust, Southampton, UK; Medical Research Council Lifecourse Epidemiology Unit, University of Southampton, Southampton, UK; NIHR Southampton Biomedical Research Centre, University Hospital Southampton NHS Foundation Trust, Southampton, UK; Medical Research Council Lifecourse Epidemiology Unit, University of Southampton, Southampton, UK; NIHR Southampton Biomedical Research Centre, University of Southampton and University Hospital Southampton NHS Foundation Trust, Southampton, UK; Medical Research Council Lifecourse Epidemiology Unit, University of Southampton, Southampton, UK; NIHR Southampton Biomedical Research Centre, University of Southampton and University Hospital Southampton NHS Foundation Trust, Southampton, UKInstitute of Developmental Sciences, University of Southampton, Southampton, UK

**Keywords:** obesity prevention, systems thinking, public health, policy, local authority, qualitative

## Abstract

**Aims:**

A whole systems approach to tackling obesity has been recommended by Public Health England for several years. This qualitative study aimed to investigate whether systems thinking is reflected in local authority plans and strategies to tackle obesity, using the leverage points for intervention in a complex system, as a framework.

**Method:**

We sought to identify obesity strategies/plans for Southampton and 19 other local authority comparators (based on children’s services and Office for National Statistics data). A healthy weight strategy was available for 10 local authorities and a qualitative document analysis was undertaken. The policy actions proposed in the plans were coded against the leverage points for intervention in a complex system and themes were developed to characterise interventions in each category.

**Results:**

A majority of actions included in the plans were categorised as ‘Numbers, Constants and Parameters’ which reflect downstream measures. However, there were examples of actions that could act on higher leverage points. In addition, some local authority plans included interventions that could act on 10 of the 12 leverage points suggesting incorporation of systems thinking.

**Conclusions:**

Some local authority plans to tackle obesity do reflect systems thinking when viewed through the lens of the leverage points for intervention in a complex system. Interventions at higher leverage points should be prioritised by public health decision-makers, especially in a climate of competing agendas and limited resources.

## Introduction

Obesity is a prominent UK public health concern, costing the National Health Service (NHS) annually about £6.1b in direct and an estimated £27b in wider societal pre-pandemic costs.^1^ Driven by ‘societal dynamics’ comprising urbanisation, food systems (including marketing and food culture), cultural norms, sedentary jobs and an environment which encourages sedentary behaviour,^2^ obesity is an emergent property of economic systems which prioritise Gross Domestic Product (GDP) growth without consideration of the adverse effects on health and the environment.^3^

Overweight and obesity in children and adolescents is a predictor of excess weight in adulthood.^4,5^ A study in the US evaluating life-course trajectories of obesity showed that at any given age more people are obese than in earlier generations and are experiencing obesity for longer, which may impact on the onset of obesity-related diseases like type 2 diabetes.^6^ For obesity prevention, the evidence points to the critical importance of intervening in childhood as well as prioritising treatment.^7^ The notion that obesity occurs due to excess calorie consumption in relation to energy expenditure, has long been considered too simplistic. It is now clear that environments during development and early life influence the way an individual responds to later environmental and physiological challenges, emphasising the importance of a life-course approach to preventing obesity.^8,9^

The socio-ecological model of health proposed over three decades ago sought to shift attention from individual to environmental causes of behaviour.^10^ The model drew attention to several levels including institutional, community and public policy in relation to individual health behaviour. However, it did not take into account causes of poor health such as economic inequalities, discrimination, unemployment, exposure to toxins and genetic predisposition.^10^ In contrast, complex adaptive systems models allow consideration of the dynamic reciprocal relationships between various factors at different levels and provide a means through which to view complex public health problems such as obesity.^11^

In England, in recognition of the poorer outcomes seen for people with excess weight, who become infected with coronavirus, the most recent government strategy proposed an expansion of weight management services and a ban on TV and online advertising of foods high in fat, sugar and salt before 9 pm and restrictions on their promotion by location and price.^12^

Local authorities also have a role to play in childhood obesity prevention. The UK government has devolved many public health responsibilities to local authorities (LAs), such as the development of the local urban environment, local transport and licensing powers.^13^ Influencing LA policies may be key to promoting a healthy weight environment which in turn could impact on obesity. However, tackling obesity including childhood obesity is not mandatory for LAs.^14^

The term ‘systems thinking’ describes a way of considering how individuals, groups, services and organisations interconnect and influence each other.^15^ In the context of obesity, a whole systems approach acknowledges the influences operating at different levels, by different actors.^16^ Systems thinking helps to reveal the characteristics and relationships between different elements of a system and to bring to light potential solutions acting across it.^16^ In the original work to identify points at which to intervene in a complex system, Meadows identified 12 ‘leverage points’.^17^ She argued that many of these are counterintuitive, and in practice, most interventions focus on the least powerful (regarded as ‘downstream’) as they are seen as the easiest points at which to intervene.^17^

Systems science considers the complexity and dynamic relationships between different components of a system as well as the context of the system.^18,19^ In 2014, the whole systems approach to obesity was recommended by Directors of Public health and PHE (Public Health England functions are now included in OHID-Office for Health Improvement and Disparities), this was well before the PHE guidance *Whole systems approach to obesity-a guide to support local approaches to promoting a healthy weight* was published in 2019.^20,21^ It is an approach that responds to complexity by allowing stakeholders to develop a shared understanding of the challenge in the local system and identify opportunities for change.^21^ The aim of the present study was therefore to investigate whether LA plans/strategies to tackle obesity and childhood obesity (through the broad frame of obesity prevention), reflect systems thinking.

## Methods

### Framework for analysis

This qualitative study was undertaken using Meadows’ 12 leverage points at which to intervene in a complex system.^17^ A number of frameworks or models have been derived from Meadows’ original work, including the Intervention Level Framework (ILF) which has five intervention levels (paradigm, goals, system structure, feedback & delays, and structural elements).^22^ Also, the Action Scales Model developed by Leeds Beckett University with four intervention levels (system beliefs, system goals, system structures and events).^21^ All models have their limitations, and in particular, the condensed models may not capture important differences between interventions operating at different levels. For example, ‘system structure’ encompasses physical structures, relationships and information flows, which are likely to require different types of intervention. Therefore, in this study, the leverage points originally described by Meadows (Table 1) were used as a framework to allow a more nuanced analysis. The leverage point ‘Transcending paradigm’ was not included as part of the framework; for this analysis, it was considered an unlikely point of intervention at local government level.

**Table 1:** Summary of the 12 leverage points to intervene in a complex system framework^17^.

Intervention level	Description
Transcending paradigm	This represents one’s view of reality and is unlikely to be influenced at local government level.
Paradigm	‘Shared unstated assumptions’ and ‘deepest held beliefs’ that are the hardest to change within a society, as it requires individuals to look at the system. Paradigms pave the way for a system’s structure which includes ‘goals’ and ‘rules’ (titles of other leverage points). Potential actions to change a paradigm include highlighting flaws with the current paradigm.
Goals	The goals of a system are considered an important point for intervention. Actors operating in a system may not be aware of the goals and they are likely to be changed by those in power.
Self-organisation	Self-organisation is a key factor in a system’s resilience. For a system to continue to exist, it must evolve as contexts change. This intervention point is concerned largely with encouraging variability and diversity in the system.
Rules	Rules include laws, regulations and incentives which help to structure a system. A fundamental point made by Meadows is that rules put in place must be made in the context agreed by a range of sectors in society to ensure they are fair and do not benefit some to the exclusion of many.
Information flows	This involves providing timely information to relevant actors which was not previously available to them, to support a course of action which may not have occurred without that information.
Reinforcing feedback loops	These are described as ‘the source of growth, explosion, erosion and collapse’ where more generates more. For example a high interest rate on higher savings, where a bigger bank balance accumulates more interest which in turn leads to more interest.^17^ Meadows points out that there are few reinforcing loops and the emphasis for the leverage point is slowing the growth.
Balancing feedback loops	These are the feedback loops that self-correct impacts on the system, often called ‘thermostats’. Balancing loops may be inactive a lot of the time and come into play at other times such as emergencies. An example could be tax on fuel emissions which are triggered once emissions reach a certain level. At this leverage point, an intervention would strengthen the feedback loop or prevent it from being weakened.
Delays	This leverage point focuses on timely information and timely responses. If feedback occurs too soon the system may overreact, if it receives feedback too slowly the system may become damaged.
Stocks and flows	Refers to physical structures in a system which may be difficult and costly to change. Intervening at this point would include building the appropriate structures at the start.
Buffers	This describes a physical entity, having enough of which helps to preserve a system.
Numbers, constants and parameters	This includes changes in people/staff and skills or having parameters for existing activities. Interventions at this point are unlikely to change the behaviour of the system unless they influence other higher leverage points. This is the commonest point of intervention.

### Selecting local authorities

As the lead researcher for this study is a Public Health Practitioner at Southampton City Council, Southampton and 19 other statistical neighbours were chosen as the LA research sites. LA statistical neighbours are based on demographic similarities (in this case, using children’s services data and Office for National Statistics (ONS) data for 2018); statistical neighbours are used by Southampton City Council to benchmark National Child Measurement Programme data and therefore were considered suitable for this analysis. Statistical neighbours and ONS comparators for Southampton are routinely updated by Southampton City Council.^23^

To identify LA obesity strategies/plans, Internet and LA website searches were undertaken using the following search terms: ‘healthy weight’, ‘obesity’ and ‘childhood obesity’. For each search term, the first 100 results from the LA website were reviewed for up-to-date local plans, policies and strategies for tackling obesity in their area. In addition, the Director of Public Health for each LA was contacted by e-mail to request a copy of their plan. The local authority plans and strategies analysed were developed before the release of the PHE guidance on the whole systems approach.

### Analysis

The first author undertook a document analysis of local government plans to tackle obesity using deductive thematic analysis as outlined by Braun and Clarke.^24,25^ This study focussed on the priorities, goals and actions to tackle obesity. Local data, descriptions of the causes of obesity and information on the wider context of the local area were excluded from the analysis. The data were extracted into a separate document and categorised into one of the categories representing the system leverage points for intervention as defined by Meadows (‘Transcending paradigm’ was not included).^17^ Each of the interventions/actions was given a code to capture its key features. Similar codes were combined and condensed. The themes developed under each category described the aim or nature of the interventions within that theme. The actions were interpreted in terms of how they aimed to change the system, for example, to change the physical environment, address socio-economic challenges, influence networks (at the higher leverage points) or target individual behaviour. This was consistent with methods used by other researchers.^22,26^ The coding, the coding frame and excerpts of the plans and strategies were shared and discussed with co-authors over several meetings during the coding process to agree the approach. The themes were also reviewed and discussed with co-authors to ensure that they reflected the data and to help improve the rigour of the analysis.^27^ NVIVO 12 Pro was used to code the data.

## Results

### Local authorities

The search for obesity plans took place during January to March 2019. Ten LAs had a healthy weight strategy, either shared by the Public Health team or accessible online. Two of the authorities provided two strategy documents (e.g. separate strategies to address physical activity and the food environment) which they collectively considered a plan to tackle obesity. Some plans were draft documents and the time period covered by the plans varied between 3 and 10 years. Not all documents included in this analysis were publicly accessible therefore the plans and excerpts taken were anonymised and names of cities were removed.

Four LAs confirmed that there was no plan in place and for six LAs a plan was not found through the Internet search and the LAs did not respond to emails requesting a copy of their plan, therefore it could not be determined whether an obesity plan was in place.

The results in Table 2 show which of the 11 leverage points (not including ‘Transcending paradigm’) featured in each LA plans to tackle obesity.

**Table 2:** Leverage points for intervention in local authority strategies to tackle obesity.

Local authority	Paradigm	Goals	Self-organisation	Rules	Information Flows	Reinforcing balancing loop	Balancing loops	Delays	Stocks and flows	Buffers	Numbers
LA1	*	*	✓	✓	✓		✓		✓	✓	✓
LA2	*	✓	✓	*	✓				✓	✓	✓
LA3a	*	*	✓	✓	✓						✓
LA3b	*	✓		*				✓			✓
LA4	*	✓	✓	✓	✓	✓	✓		✓	✓	✓
LA5	*	✓	✓	✓	✓	✓	✓		✓	✓	✓
LA6	*	✓	✓		✓			✓	✓		✓
LA7a	✓	✓	✓	✓	✓	✓	✓			✓	✓
LA7b	*	*	✓	*	✓	✓			✓	✓	✓
LA8	*	✓	✓		✓			✓	✓	✓	✓
LA9	*	*		*	✓				✓	✓	✓
LA10 Southampton	*	✓	✓	✓	✓				✓		✓

LA: local authorities.

Paradigm- * a new paradigm is described but problems with the current paradigm are not directly highlighted. Paradigm- ✓ if problems with the current paradigm are described.

Goals- * where goals are provided but do not address problems with the paradigm. Goals- ✓ if they address the paradigm.

Rules- * if they are voluntary parameters. Rules- ✓ include planning documents or regulatory functions.

The majority of interventions identified in the plans/strategies acted on the least powerful leverage point ‘Numbers, Constants and Parameters’ (Table 2). However, a range of interventions acting at higher leverage points were identified, and two LA plans had examples of interventions which could act on 10 of the 11 leverage points looked at as part of this study. This suggests that systems thinking was incorporated in some LA plans to tackle obesity. However, for ‘Paradigm’ most strategies set out a vision for a better paradigm rather than highlighting flaws in the existing paradigm and setting ‘Goals’ to correct them. Few strategies included interventions that operated on the ‘Delays’, ‘Balancing Feedback Loops’ and ‘Reinforcing Feedback Loops’ leverage points.

For each category, between two and six themes emerged which describe the intervention or action. Table 3 gives examples of interventions at LA level to tackle obesity and illustrates how they were categorised. It also highlights the challenges of categorising local interventions in relation to a complex system. Specific issues for each leverage point are discussed below.

**Table 3:** The leverage points to intervene in a complex system (not including Transcending paradigm) and local authority examples of interventions to tackle obesity.

Leverage point	Theme	Description	Examples of interventions (excerpts from local plans/strategies)
2. Paradigms – ‘shared unstated assumptions’ and ‘deepest held beliefs’ that are the hardest to change within a society, as it requires individuals to look at the system. Potential actions to change a paradigm include highlighting flaws with the current paradigm.	2a. Create a culture, environment and opportunities to promote healthy weight	Aspiration to create local culture and opportunities to promote and enable health, healthy weight, healthy eating and physical activity.	X city will be an active, healthy city where residents maintain a healthy weight from childhood through adult life and into older age. (LA4)
	2b. Make the issue everybody’s business	Broad statement/aspiration to make the issue everybody’s business.	Make healthy weight a priority for all: Ensure all partners at all levels view healthy weight as a priority and are actively engaged in supporting and contributing to increasing our healthy weight population (LA6)
	2c. Target those in greatest need	Broad statement/aspiration to target those in greatest need.	The aim is to create a ‘healthy weight’ environment where healthy choices are the easy choices for children; as well as ensuring early intervention targets those in greatest need. (LA10)
	2d. Tackle issues and risks caused by the current paradigm	Highlights current wider system problems that are contributing to the issue. Welfare reforms and austerity. **Directly highlights flaws in the current paradigm**	Welfare reform – the welfare system is increasingly failing to provide a robust last line of defence against hunger. Changes to the welfare system, performance of the benefits system and the increasing use of sanctions have contributed to increases in demand for emergency food assistance both locally and nationally. There is concern that the roll out of Universal Credit could worsen the situation by putting claimants into debt and rent arrears and by disrupting the allocation of free school meals by removing the current eligibility triggers. (LA7a)
3. Goals – the goals of a system are considered an important point for intervention. Actors operating in a system may not be aware of the goals and they are likely to be changed by those in power.	3a. Influence national and regional agenda	Aim to work with national government or influence the national agenda.	Lobbying – Increase in fiscal, food production and marketing measures that support children to be a healthy weight. (LA3a) To influence the regional and national agenda to promote healthy weight. (LA2)
	3b. Give children the best start.	Aim for children to have a healthy weight, giving children the best start. Families supported by a competent work force.	To tackle overweight and obesity effectively we need to adopt a life course approach – from pre-conception through pregnancy to preparing for older age. (LA1) To give all children the best start in life and halt the rising tide of childhood obesity across the city; (LA4)
	3c. Provide weight management support and campaigns	Aim to address obesity providing weight management support and signposting.	To offer effective support for families and individuals who want to lose weight. (LA2) Offer effective support for children and adults who want to lose weight (LA5)
	3d. Tackle wider determinants – crime, unsocial behaviour, poverty, inequalities and obesogenic environment	Goals to address wider/environmental determinants of health and tackle poverty, crime and narrow inequalities. **These goals aim to change the paradigm**	The current healthy weight inequalities gaps will be narrowed. The causes that put particular groups of children at higher risk of an unhealthy weight will be addressed.(LA3b) Wherever possible the proposed actions in the strategy will seek to support wider outcomes related to food including mitigating the worst effects of poverty, strengthening the local economy, reducing carbon emissions, increasing the resilience of our food supply networks and promoting social cohesion and general wellbeing through food. (LA7a)
4. Self-organisation – self-organisation is a key factor in a system’s resilience. For a system to continue to exist it must evolve as contexts change, which creates further complexity. This intervention point is concerned with evolving a system and adding to its resilience.	4a. Food partnerships	Food partnerships-stakeholders brought together to improve the local food environment.	The x city Food People is a network of food growers, composters, buyers, cooks and eaters passionate about a positive healthy food culture for the city. It is led through the third sector, with members currently hailing from a range of providers and programmes across the city. (LA4)
	4b. Healthy weight partnerships	Healthy weight partnerships – stakeholders brought together to oversee implementation of a healthy weight strategy.	Establish an accountable Healthy Weight Alliance to maintaining partnership action across the Healthy Weight Strategy action plan. (LA8)
	4c. Physical activity partnerships	Physical activity partnerships – stakeholders brought together to increase physical activity.	Increase opportunities for physical activity in our daily lives, reducing sedentary behaviour – (delivered through Fit for Life strategy and partnership). (LA1)
5. Rules- include laws, regulations and incentives which help to structure a system.	5a. Local policies, standards and best practice for healthy environments delivered through council policies, contracts and services	Using environmental health and the Healthy Weight Declaration to provide standards to promote healthy weight. Weight management standards in service contracts and policies for local partnerships.	Use council services such as environmental regulations, licensing and city centre management to engage private industry with responsible retailing and healthier food guidance (LA7a) X city Food Partnership is developing a Food ethos in regards to commercial partnership which can offer good practice for x City Council. (LA3a)
	5b. Planning mechanisms, principles and standards for external and indoor space	The Local Plan and other planning documents to restrict takeaways, impose parking standards and improve internal space standards. **These are examples of LA interventions that could be considered as acting on the rules leverage point. They are not rules in the strictest sense.**	Supplementary Planning Document on hot food takeaways drafted by City Development, consultation complete . . .x city Design Wellbeing group meet regularly and are developing principles for planning (LA3b) Implement internal space standards for new dwellings (to ensure adequate kitchen and dining space) (LA10)
	5c. Voluntary healthy food standards in settings and venues.	Healthy schools, healthy early years, UNICEF breast feeding, healthy workplaces, standards for vending machines.	Healthy Schools Programme – whole school approach to promoting healthy weight Healthy Children’s Centre standards. (LA5) Partners in the city have achieved or are working towards UNICEF Baby Friendly Initiative (BFI) full accreditation. (LA4)
6. Information flows – this involves providing timely information to relevant actors which was not previously available to them, to support a course of action which may not have occurred without that information.	6a. Individual assessment	Physical fitness assessments and NCMP measurement and feedback.	All children in reception and year 6 are weighed and measured as part of the NCMP. Families are informed if their children are overweight and recommended to attend a Tier 2 family weight management programme. (LA4)
	6b. Community insights	Community insights. Understanding influences on food choice and lifestyle behaviours.	Undertake public consultation to gain insight into how local residents would like the council to use its place shaping powers to influence the food environment and support healthier food choices. (LA7a)
	6c. Evaluating services/provision	Evaluating services and provision in the public sector, including weight management services and food provision.	The Standard Evaluation Framework for Weight Management Interventions will be used to guide the evaluation of all commissioned interventions. (LA5)
	6d. Influencing other departments, sectors, leaders and wider agendas	Gathering information/auditing to influence others. Influencing through training others including licensing, planners, developers, partners, leaders. Influencing the regional and national agenda.	Commissioned insight to understand why there are a high number of adult pedestrian road injuries in X city centre. This informs the current Adult Pedestrian Casualty Reduction programme . . . needed to encourage modal shift to walking (and cycling). (LA4) Developing and delivering group educational and leadership workshops for senior managers across the City and from different organisations to ensure ‘buy-in’. . .of promoting an active workforce – starting with Move More stakeholders. (LA7b)
7. Reinforcing feedback loops – described as ‘the source of growth, explosion, erosion and collapse’ where more generates more. The intervention for this leverage point is to slow the growth.	7a. Reduce debts, poverty and food poverty	Reduce debts, improve financial ability to access healthy food.	Tackle poverty and deprivation by getting more local people into good jobs. (LA5)
	7b. Limit exposure to junk food	Limit exposure to unhealthy food in public sector settings and events.	Ensure food and drinks provided at public events include healthy provisions, supporting food retailers to deliver this offer. (LA4l)
	7c. Reduce and limit crime and remove cues that limit physical activity	Reduce crime or fear of crime. Remove cues that limit physical activity	Developing a programme of work to review and replace cues from the physical and social environment that re-enforce the message that physical activity is not important. For example; cycle paths that are not connected, not sufficiently wide or give way to motorists unnecessarily OR streets that are littered with ‘no ball game’ signs or city centres designed around car access rather than active travel access (i.e. lots of car parks and no safe cycle storage or changing facilities). (LA7b)
8. Balancing feedback loops – mechanisms that self-correct the impact on the system. Balancing loops may be inactive a lot of the time and come into play at other times such as emergencies.	8a. Limit unhealthy snack and food outlets	Limit adverts for junk food in council controlled places and events. Subsidies/altered business rates to promote for healthier food.	Limiting exposure to cheap and appealing calorie-dense, nutrient-poor food in the wider environment and restricting opportunities for the marketing of this type of food (particularly in places where the council has some control or influence). (LA4)
	8b. Protect outdoor green spaces	Protect outdoor spaces and sports pitches from development.	Ensuring future development in x city creates/maintains healthy communities through the provision and protection of open space for physical activity and food growing, where possible and through restricting unhealthy uses (e.g. takeaways) in areas where it is deemed necessary. (LA5)
9. Delays–this leverage point focuses on timely information and responses.	9a. Timely health impact assessments	Timely strategic assessments.	Ensure Health Impact Assessments are carried out in a timely and appropriate way (LA8)
	9b. Timely identification of those with excess weight	Reducing delays for people who need services.	Children who are an unhealthy weight will be identified early and supported.(LA3b) Capitalise on early intervention and treatment: Support those outside the healthy weight category to become and maintain a healthy weight through a range of evidence-based interventions (LA6)
10. Stocks and flows – refer to physical structures in a system.	10a. Development of the local plan and SPDs for a healthy weight environment	Contribute to the local plan, supplementary planning documents (SPDs) for building design to enable environments for physical activity and healthy eating.	Ensure principles of planning for healthy weight environments are embedded in the new Local Plan. (LA10) Design and re-orientate buildings so that they promote opportunities for active living and at the same time reduce sedentary behaviour (e.g. enhancing signage to the stairs and improving the quality of stairwell environments). (LA7b)
	10b. Improve walking and cycling infrastructure and road safety	Develop cycling walking infrastructure and connectivity and improve road safety.	As part of an environmental approach to increasing physical activity the ‘cycle-ability’ and ‘walkability’ of x city will be improved via a programme of investment under the proposed banner ‘Routes to Activity’. (LA7b)
	10c. Improve open and green infrastructure	Improve parks, open spaces, remove unnecessary signage (no ball games) and car- parking. Connected green spaces and playgrounds.	Develop a well-connected multifunctional network of green infrastructure. Review and improve availability of green spaces and playing pitches as well as safeguard against the loss of open space and recreational facilities. (LA1)
11. Buffers – this describes a physical entity, having enough of which helps to preserve a system.	11a. Increase access to space and facilities for physical activity	Enable easier access to physical activity through increased availability of open green spaces, free physical activity or community facilities.	By making more space available for people to grow their own produce, designing buildings which encourage stair use and urban planning which facilitates active transport all help increase access to healthy food and opportunities to be more physically active. (LA8) Support schools to be community hubs providing access to their facilities in their local community to raise awareness and encourage families to be more active. (LA1)
	11b. Increase availability of affordable healthy food and drink	Increase availability of healthy food for target groups, increase access to free drinking water. Incentivise healthy food retailers. Increase: allotments for food growing, healthy vending machines, UNICEF venues.	Use of incentives/subsidies/differential business rates to attract healthier food retailers into areas where they are lacking. (LA7a) Voucher or subsidy schemes for individuals on low incomes or in deprived neighbourhoods to incentivise the purchasing of fruit and vegetables. (LA4)
	11c. Promote the living wage and welfare	Work to improve economic circumstances of residents in need.	Promoting the living wage and equal pay for women with local businesses, as a basis for good employment. (LA4)
12. Numbers – this includes changes in people/staff and skills or having parameters for existing activities. Interventions at this point are unlikely to change the behaviour of the system unless they influence higher leverage points.	12a. Community healthy eating initiatives	Cookery programmes. Growing schemes and community healthy lifestyle programmes.	Community-based cookery programmes for example: X City Farm cookery programme for mental health service users. Cookery programmes for young adults and older people in supported housing and volunteering projects to improve cooking and food growing skills (LA1)
	12b. Family and general weight management advice, programmes, initiatives and campaigns	Children and adult weight management programmes. Children’s healthy lifestyle activities. HENRY/Breastfeeding/ weaning/Postnatal programmes. Healthy start scheme promotion. National and local campaigns, signposting.	Interventions will be available to children and adults who are above a healthy weight. We will also widen access by offering a greater range of interventions at differing intensities that reflect level of need. (LA7a) Provide opportunities to engage the public in health promoting behaviours. Tailor information and support to groups at higher risk of overweight or obesity through activities provided by the Behaviour Change Programme. (LA2)
	12c. Initiatives to promote healthy settings	Projects/programmes to promote healthy early years settings, healthy school environments, healthy workplaces. Daily mile, school sport. Supporting resources and toolkits.	Provide advice and guidance for settings including schools, care settings and home care providers to introduce measures that encourage healthy eating, prioritising those with highest rates of overweight and obesity. (LA7a) We would like to ensure schools are supported to provide nutritionally healthy meals using this locally produced toolkit. (LA3b)
	12d. Initiatives to increase physical activity	Deliver/promote initiatives for reducing sedentary behaviour/ increasing physical activity/sports/recreation/use of green space. Encouraging active travel (walking and cycling), road safety. Exercise on referral.	Encourage bike-ability training for children and promoting urban cycling skills to parents including improving accessibility to equipment to enable safe cycling. (LA9) Work with partners, including leisure providers and local businesses to promote opportunities and projects which achieve a sustainable increase in physical activity among, families with young children, school aged children and young people, targeting those in greatest need (LA10)
	12e. Staff training and resources	Front line staff appropriately trained. Training and toolkits developed. Making every contact count training.	Provide brief intervention training for GPs and other front line health workers Ensure that messages about losing excess weight are consistently clear and concise by providing brief intervention training for GPs and other frontline healthcare workers involved in weight management interventions. (LA9)
	12f. Work with industry and develop guidance and incentives to promote healthy choices	Guidance for businesses/suppliers/ retailers. Develop commercial partnerships. Commercial concessions/pledges Promoting healthy choices.	Consider how commercial partnerships with the food and drink industry may impact on the messages communicated around healthy weight to our local communities. (LA4) Work with businesses and partners to promote city wide initiatives/campaigns which promote sustainable increase in physical activity (PA), makes use of green/open spaces and promotes healthy food choices (LA10)

LA: local authorities; UNICEF: United Nations International Children’s Emergency Fund; BFI: Baby Friendly Initiative; NCMP: National Child Measurement Programme; PA: physical activity.

### Paradigm

Vision statements which specified actions were considered to be interventions acting on the ‘Paradigm’. A way to intervene at this leverage point is to highlight the problems with that paradigm, Meadows notes that to intervene ‘you keep pointing at the anomalies and failures in the old paradigm’ (Wright and Meadows 2009 p164).^17^ One strategy (LA7a) directly highlighted flaws in the paradigm potentially linked to obesity, specifically in relation to food availability and policies on austerity and welfare reforms.

Most strategies set out positive aspirational vision statements for the city or residents. These statements are likely to be important to engage LA leaders. However, focussing on individuals, families and communities, moves attention away from the problems with the system and towards those affected by the system.

### Goals

‘Goals’ in strategies added detail to the vision statements. As the flaws in the paradigm were not clearly articulated in most strategies, the goals did not directly attempt to reshape or ‘improve’ the system. However, some policies did reflect the negative effects of the current paradigm and sought to address issues broadly such as inequalities, crime, poverty and the obesogenic environment (including preserving green space and improving cycle infrastructure). Overall goals were aspirational in terms of how the future could be shaped.

### Self-organisation

Evidence of ‘Self-organisation’ was apparent in most plans. The main examples were networks and partnerships set up to deliver part of the strategy/plan in relation to the food environment and promoting physical activity. They included strategic networks and networks of stakeholders and champions to promote, deliver or oversee elements of the strategy.

### Rules

Many of the interventions coded as ‘Rules’ in this analysis could technically be considered as ‘Numbers, Constants and Parameters’ as they are not rules in the strictest sense; rather they are recommended standards or parameters within which to operate.

The interventions categorised as ‘Rules’ were supplementary planning documents. These are non-statutory documents which support town planners when making development decisions. They may provide LAs with strategies to regulate the food environment. However, they are non-statutory and any decisions can be appealed.

Other interventions classed as ‘Rules’ included voluntary programmes supporting standards on nutrition and physical activity aimed at schools and workplaces. In the UK, School Food Standards are mandatory, however, monitoring mechanisms are weak and not clearly enforced.^28^

### Information flows

Examples of interventions acting on ‘Information Flows’ were found in most strategies/plans. At an individual level, this included feedback from the National Child Measurement Programme (NCMP). At a community level, it included plans to gather community insights on lifestyle choices. Interventions to influence the system included a public street audit to inform transport decisions. These ‘Information flows’ may change the course of action of the individual or group receiving the information.^17^

### Reinforcing feedback loops

Feedback is an important component of systems thinking, ‘Reinforcing Feedback Loops’ in a system can reinforce healthy or unhealthy behaviours.^29^ The aim is to slow down the feedback loop reinforcing the unhealthy behaviour. Systems diagrams for obesity were not included in LA plans, consequently interventions for ‘Reinforcing Feedback Loops’ were not identified. Therefore, community-based systems maps for obesity in the published literature were used as reference points to identify them.^30,31^

Three main types of intervention were identified which aimed to limit undesirable outcomes; first, making healthy food more available (e.g. through voucher schemes for food); second, supporting families to reduce debts, promoting the living wage and enabling access to jobs; and third, creating safe environments and removing cues that discourage physical activity.

### Balancing feedback loops

Interventions acting on ‘Balancing Feedback Loops’ should self-correct a system, suggesting an in-built mechanism to moderate an effect which can be reinforced to move the system in the desired direction.^17,29^ Examples included restrictions on the concentration of hot food takeaways and limiting developments on community green spaces. These interventions allow for development up to a point at which restrictions are triggered.

### Delays

‘Delays’ are concerned with timely inputs and responses to influence a system. Whether the intervention is to speed up or slow down the response is dependent on the desired outcome.^17^ The one example of an intervention acting at a structural level was the timely implementation of Health Impact Assessments (LA8). Several other strategies mentioned timeliness at an individual level, for early identification and treatment of obesity.

### Stocks and flows

‘Stocks and Flows’ have been described as the ‘plumbing structure’ of a system and interventions for this are considered slow and costly.^17^ Examples included actions such as ‘green space improvements’ or ‘ensure spatial planning processes support promoting a healthy weight’. The interventions were not specific, which may have been deliberate and used as a ‘catch all’ statement of intent for obesity prevention. More specific actions focused on investments in cycling and walking infrastructure.

### Buffers

Interventions acting on the ‘Buffers’ leverage point included; increasing the availability of healthy food, improving the availability of facilities for physical activity such as open and green spaces and providing a financial buffer for families. This leverage point was ranked low by Meadows in terms of influencing a system.

### Numbers, constants and parameters

A majority of the actions in the LA strategies were coded as ‘Numbers, Constants and Parameters’. There were numerous examples of interventions including developing and delivering staff training, or community education on a range of topics including Making Every Contact Count (MECC), healthy lifestyles and signposting to existing services. Interventions also included weight management support, healthy eating and physical activity initiatives.

## Discussion

This study investigated whether LA strategies/plans to tackle obesity reflect systems thinking using the leverage points for intervention proposed by Meadows.^17^ The analysis revealed the majority of interventions could be categorised as ‘Numbers Constants and Parameters’. However, a range of practice-based interventions which could act on most of the different leverage points were also identified in some plans, suggesting that systems thinking was considered by some LAs. Viewing interventions through the lens of leverage points highlighted the limitations of many interventions in a local context, but the analysis also revealed potential synergies between them.

More interventions were coded as ‘Numbers, Constants and Parameters’ than any other leverage point. This is in accordance with previous analyses of national and local policies which showed that most strategies focussed on downstream measures to improve lifestyle behaviours through health education.^14,22,32^ Although this leverage point is considered the least potent, it could nonetheless be important if the intervention triggers action at a higher leverage point.^17^ Finegood provided an example where information about the adverse effects of secondhand smoke led people to demand ‘Rules’ on smoke-free spaces.^29^

The three leverage points defined by Meadows as offering the best opportunities for change are also the most difficult points at which to intervene, namely ‘Transcending Paradigm’, ‘Paradigm’ and ‘Goals’. This analysis provides examples of how interventions could act on the ‘Paradigm’ and ‘Goals’ but may not change the system at LA level (‘Transcending Paradigms’ were not included in the analysis). More research is therefore required to understand if and how interventions at these points could change the system to tackle obesity at a local level. For the remaining leverage points, Meadows suggests interventions should focus on how to prevent the system from producing undesirable outcomes.^17^ Actions to change the paradigm include identifying its flaws.^17,33^ For obesity, this could be through highlighting the influence of economic and political environments and social inequalities^29^ and the links to powerful private sector actors.^33^ This was observed in one strategy. Not clearly articulating the flaws of the current system may lead to ‘Goals’ which do not directly address the problems in the system, reducing the likelihood of actually changing it.

Flaws with the paradigm may be laid out in other relevant documents including independent Director of Public Health annual reports (e.g. Southampton 2017).^34^ Nevertheless, highlighting the flaws in the local paradigm may be contentious in local councils which are political organisations, where councillors decide on the policy framework and the officers role is to support its delivery.^35^ However, this may be an important role that can be undertaken by experts and other leaders in the system.

Evidence of interventions that acted on the ‘Self-organisation’ leverage point focussed on setting up food/nutrition or physical activity partnerships to address obesity as part of local strategies. However, self-organisation should not be directed externally and should emerge (in response to a need) and regulate itself.^36,37^ It is possible that public health driven partnerships could lead to the development of ‘organic’ community-led networks (e.g. community food networks) which would have greater fidelity to the notion of self-organisation.

The impact of interventions acting on the ‘Rules’ leverage point may be limited in local settings where they cannot be enforced, for example physical activity standards in schools. Therefore, consideration should be given to how a majority of the target audience/settings could be encouraged to adopt the ‘Rules’. This could be through the rules becoming embedded through appropriate incentives to secure widespread compliance. Rules could also include informal structures such as customs, taboos and codes of conduct which can be deeply embedded in society and influence or limit action.^38^

Synergies between leverage points were also revealed, for example acting on the ‘Buffer’ to increase the availability of inexpensive, healthy food for people on low incomes, may inhibit a ‘Reinforcing Feedback Loop’ which normally causes people on low incomes to consume poorer quality diets. In addition, some interventions may work simultaneously on different leverage points; for example a supplementary planning document may contribute to local ‘Rules’ and could act on ‘Balancing Feedback Loops’ if it aims to slow proliferation of takeaways after a set parameter is reached. Other researchers have highlighted the importance of understanding the interdependencies of different interventions but more research is required to understand how this works in practice.^38^ The analysis also highlighted that while leverage points such as the ‘Paradigm’ and ‘Goals’ of the system are unlikely to be changed locally, ‘Stocks and Flows’, a lower leverage point may be more readily influenced through the planning powers of LAs.^39^

The collaborative approach to systems thinking provided by systems dynamics requires the bringing together of stakeholders to describe the system (by producing a systems diagram) and identifying opportunities to intervene. However, in practice, points to intervene will be determined by collaborators who choose to participate and have the resources to do so.^40^ Consequently, there is a risk that this approach may be biased and only tackle a part of the system.^17^ The 12 leverage points framework could provide useful prompts for public health teams to help ensure a range of interventions including those acting on higher leverage points are considered. Viewing interventions through the lens of the leverage points to intervene may increase understanding of how a range of interventions could reshape a system as well as highlight potential constraints for achieving a system change. This is especially important where resources, as well as expertise in systems dynamics, may be limited and this analysis revealed practice-based examples of how interventions at these leverage points may work to address obesity at a local level.

There is no single system that causes obesity, but there are systems which may contribute, for example the economic system, the food system, the transport system and the welfare system.^3^ Understanding how they influence obesity at a local level may help to identify different points at which to intervene. Complex systems will have feedback loops and information flows that lead to both desirable and undesirable outcomes, which are in some way linked.^17^ Therefore, the aim is to strengthen the parts of the system that work, and weaken the undesirable parts. Meadows notes that ‘systems can’t be controlled but they can be designed and redesigned’ (Wright and Meadows 2009, p 169).^17^ It may be that the causes of optimum weight need to be conceptualised as a complex system. This system would change over time and through the life-course and the functions contributing to healthy weight could be strengthened.

## Strengths And Limitations

This is the first study to provide a unique insight into how interventions aimed at tackling obesity in LAs could reflect systems thinking. It involved the analysis of strategies from 10 of the 20 LAs, selected for their demographic similarities (the remaining 10 local authorities either did not have a plan or it was unknown whether a plan was in place). Although the LAs included were from a wide geographic area, the plans may not reflect practice in other LAs in England.

In addition, the search terms used may have missed other LA strategies relevant to obesity prevention. However, this was a novel approach to view local interventions and should be seen as a starting point for the analysis of a broader set of local authority plans and strategies in order to develop the evidence base on how interventions could act on leverage points to change a system.

The analysis was undertaken before the release of guidance on a whole systems approach and it does not provide evaluative evidence about how interventions implemented by LAs change a complex system. However, viewing interventions through the lens of the 12 leverage points, as described in this study, could support LAs in prioritising interventions more likely to change the system.

While the strategies/plans were reviewed, and interventions categorised by one researcher, the approach taken to assign the interventions was discussed with other researchers with reference to Meadows’ framework.^17^

The Meadows’ framework was used in this study, although several other more recent frameworks derived from this have clustered the leverage points to ‘operationalise’ systems thinking.^22,38^ However, understanding the intended function of an intervention is crucial during implementation and using aggregated models make this more difficult.

## Conclusion

Many of the interventions to tackle obesity in LAs are downstream measures influencing the least important leverage point at which to intervene in a complex system.^17^ However, this analysis revealed practice-based examples of interventions that could work upstream on higher leverage points. Using the whole systems approach to identify opportunities for intervention should be followed by considering how interventions acting on higher leverage points could be prioritised. This study highlights examples of interventions planned by LAs, however, more research is required to evaluate how these interventions could change a system in practice.

Given that, systems thinking is increasingly important in public health practice, training and professional development opportunities to build deeper understanding of these complex concepts should be considered.
